# Nomogram for predicting post-therapy recurrence in BCLC A/B hepatocellular carcinoma with Child-Pugh B cirrhosis

**DOI:** 10.3389/fimmu.2024.1369988

**Published:** 2024-05-10

**Authors:** Wenying Qiao, Shugui Sheng, Yiqi Xiong, Ming Han, Ronghua Jin, Caixia Hu

**Affiliations:** ^1^ Interventional Therapy Center for Oncology, Beijing You’an Hospital, Capital Medical University, Beijing, China; ^2^ Beijing Key Laboratory of Emerging Infectious Diseases, Institute of Infectious Diseases, Beijing Ditan Hospital, Capital Medical University, Beijing, China; ^3^ Beijing Institute of Infectious Diseases, Beijing, China; ^4^ National Center for Infectious Diseases, Beijing Ditan Hospital, Capital Medical University, Beijing, China; ^5^ Changping Laboratory, Beijing, China

**Keywords:** hepatocellular carcinoma (HCC), recurrence free survival (RFS), random survival forest (RSF), LASSO regression, multivariate Cox regression, nomogram

## Abstract

**Introduction:**

This study conducts a retrospective analysis on patients with BCLC stage A/B hepatocellular carcinoma (HCC) accompanied by Child-Pugh B cirrhosis, who underwent transarterial chemoembolization (TACE) in combination with local ablation therapy. Our goal was to uncover risk factors contributing to post-treatment recurrence and to develop and validate an innovative 1-, 3-, and 5-year recurrence free survival (RFS) nomogram.

**Methods:**

Data from 255 BCLC A/B HCC patients with Child-Pugh B cirrhosis treated at Beijing You’an Hospital (January 2014 - January 2020) were analyzed using random survival forest (RSF), LASSO regression, and multivariate Cox regression to identify independent risk factors for RFS. The prognostic nomogram was then constructed and validated, categorizing patients into low, intermediate, and high-risk groups, with RFS assessed using Kaplan-Meier curves.

**Results:**

The nomogram, integrating the albumin/globulin ratio, gender, tumor number, and size, showcased robust predictive performance. Harrell’s concordance index (C-index) values for the training and validation cohorts were 0.744 (95% CI: 0.703–0.785) and 0.724 (95% CI: 0.644–0.804), respectively. The area under the curve (AUC) values for 1-, 3-, and 5-year RFS in the two cohorts were also promising. Calibration curves highlighted the nomogram’s reliability and decision curve analysis (DCA) confirmed its practical clinical benefits. Through meticulous patient stratification, we also revealed the nomogram’s efficacy in distinguishing varying recurrence risks.

**Conclusion:**

This study advances recurrence prediction in BCLC A/B HCC patients with Child-Pugh B cirrhosis following TACE combined with ablation. The established nomogram accurately predicts 1-, 3-, and 5-year RFS, facilitating timely identification of high-risk populations.

## Introduction

In 2020, primary liver cancer ranked as the sixth most common malignancy worldwide and the third leading cause of cancer-related deaths (1). Hepatocellular carcinoma (HCC) accounts for 75%-85% of primary liver cancer cases (2). While surgical resection offers an effective treatment for many HCC patients (3), in China, over 80% of HCC cases stem from hepatitis B virus (HBV) infection, and a substantial number of these patients also suffer from varying degrees of liver cirrhosis. This complicates surgical resection and increases the likelihood of postoperative complications due to compromised liver function (4, 5).

The Child-Pugh scoring system plays a pivotal role in evaluating liver function, which scores patients based on their degree of jaundice, ascites, hepatic encephalopathy, serum albumin levels, and coagulation function, and divides them into three classes: A, B, and C (6). Child-Pugh A HCC patients typically present the most ideal conditions for undergoing surgical resection, with lower surgical risks and a more favorable anticipated postoperative recovery due to their relatively good liver function (7, 8). In contrast, HCC patients with Child-Pugh C liver cirrhosis exhibit more severe impairment of liver function. Although surgical resection may not be the preferred option in these cases, liver transplantation is considered a feasible treatment choice in the absence of significant risk factors (9). While for HCC patients with Child-Pugh B cirrhosis, there is currently a lack of publicly available and explicit treatment recommendations. These patients, whose liver function is moderate compromised, often do not qualify for surgical resection or local ablation alone due to the high risks associated with their reduced hepatic reserve or existence of multiple tumor nodules. Traditional systemic chemotherapy may also be poorly tolerated due to its toxicity on the already compromised liver. In this context, transcatheter arterial chemoembolization (TACE) combined with local ablation therapy emerges as a promising approach for the treatment of BCLC stage A/B HCC patients, including those with Child-Pugh B cirrhosis (10, 11).. However, there is a scarcity of studies examining the application of this combined therapy in Barcelona Clinic Liver Cancer (BCLC) A/B HCC patients with Child-Pugh B cirrhosis, with a conspicuous dearth of attention on prognosis studies for this specific type of patients.

Therefore, our study tried to address this critical gap by conducting a retrospective analysis focused on identifying clinical factors linked to postoperative tumor recurrence in this specific patient group (BCLC A/B HCC patients with Child-Pugh B cirrhosis undergoing TACE combined with local ablation) through random survival forest (RSF), LASSO regression, and multivariate Cox regression. Then we developed and validated a novel recurrence-free survival (RFS) nomogram, marking a significant advancement in these patients prognostic prediction and treatment management, enabling clinicians to make more informed decisions and ultimately improve patient outcomes.

## Materials and methods

### Study population

**Figure 1:**
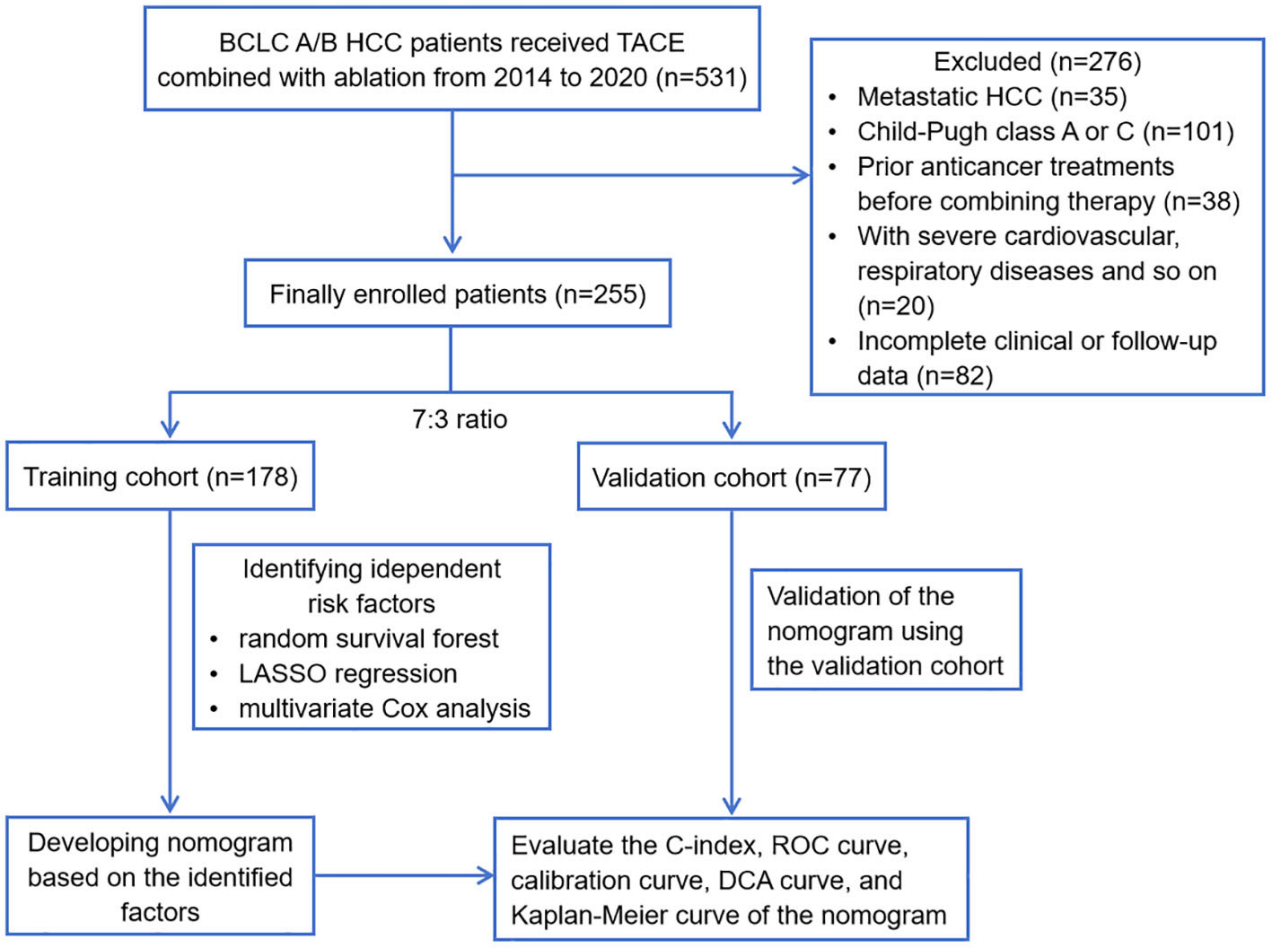
Flowchart of this study. HCC, hepatocellular carcinoma; BCLC, Barcelona Clinic Liver Cancer; TACE, transcatheter arterial chemoembolization; LASSO, least absolute shrinkage and selection operator; ROC, receiver operating characteristic; DCA, decision curve analysis.

The study was approved by the ethics committee of Beijing You’an Hospital, affiliated with Capital Medical University. All procedures adhered to the ethical standards of human experimentation at Beijing You’an Hospital and conformed to the principles outlined in the Helsinki Declaration. Taking into account the utilization of anonymized patient data, the retrospective nature and the minimal risk of the research, the committee approved a waiver for the requirement of informed consent. We firstly retrospectively reviewed the medical data of 531 consecutive patients with BCLC A/B HCC who received TACE combined with ablation treatment from the electronic medical record system of Beijing You’an Hospital, affiliated with Capital Medical University, from January 2014 to January 2020. All patients included in the study possessed a confirmed diagnosis of HCC based on the criteria established by the American Association for the Study of Liver Disease (AASLD). Then, 276 patients were excluded from the study according to the exclusion criteria: metastatic HCC (n=35); Child-Pugh class A or C (n=101); prior anticancer treatments before combining therapy (n=38); with severe cardiovascular, respiratory diseases and so on (n=20); incomplete clinical or follow-up data (n=82). Therefore, a total of 255 patients were finally included in the study. The sample size of 255 patients have reached a relatively stable size statistically, which can provide the necessary degree of freedom for analysis and ensure more accurate and reliable results. **Figure 1** displays the flowchart detailing the selection of eligible patients.

The inclusion criteria were as follows: (1) clearly confirmed HCC diagnosis; (2) received TACE combined with ablation treatment; (3) BCLC stage A or B; (4) Child-Pugh class B with liver cirrhosis; (5) complete clinical and follow-up data. The exclusion criteria were as follows: (1) metastatic HCC; (2) Child-Pugh class A or C; (3) patients who had undergone any anticancer treatments before the combination therapy; (4) accompanied by severe cardiovascular diseases, respiratory system disorders, or other serious systemic illnesses; (5) incomplete clinical or follow-up data.

### Clinicopathologic data collection

Comprehensive clinicopathologic data was systematically compiled from the enrolled patients, encompassing demographics, medical history, blood routine examinations, liver function and coagulation tests and tumor characteristics. Demographics: age and gender; Medical history: hypertension, diabetes, antiviral history, smoking history, drinking history, family history, alcoholic liver disease (ALD); Blood routine examinations: white blood cell (WBC), neutrophil to lymphocyte ratio (NLR), platelet to lymphocyte ratio (PLR), monocyte to lymphocyte ratio (MLR), neutrophil (Neu), lymphocyte (Lym), monocyte (Mon), red blood cell (RBC), hemoglobin (Hb), platelet (PLT); Liver function and coagulation tests: aspartate aminotransferase (AST), alanine aminotransferase (ALT), AST/ALT, direct bilirubin (DBIL), total bilirubin (TBIL), DBIL/TBIL, total protein (TP), albumin (Alb), globulin (Glb), Alb/Glb, gamma glutamyl transpeptidase (GGT), GGT/ALT, alkaline phosphatase (ALP), prealbumin, bile acid, uric acid, cholesterol, prothrombin time (PT), international normalized ratio (INR), thrombin time (TT), fibrinogen (Fib), Fib/Alb; Tumor characteristics: BCLC, tumor number, tumor size and alpha-fetoprotein (AFP). We would conduct subsequent analysis to screen out the most statistically significant factors for RFS among these many factors and establish an RFS nomogram.

### TACE combined with ablation procedure

All patients in this study received TACE combined with ablation therapy. This operation was performed by interventional physicians with at least 5 years of experience. TACE was administered as the initial therapeutic intervention, followed by local ablation conducted within 1 to 2 weeks subsequent to the TACE procedure. Here are the TACE procedure details. (1) Initiation: Utilizing a modified Seldinger technique, arterial puncture and catheter insertion are performed in the femoral artery. This step is critical for accessing the vascular system and navigating towards the tumor. (2) Angiography: Conducted via a catheter guided to the common hepatic artery or the mesenteric artery, angiography is essential for mapping out the tumor’s precise location, size, and quantity of lesions. (3) Embolization agents and techniques: In this stage, the selection of embolization agents is pivotal. We employ a combination of chemotherapy drugs (such as oxaliplatin, fluorouracil, or epirubicin), lipiodol, and, if necessary, gelatin sponge particles. The choice of agents is based on the tumor’s characteristics and the patient’s liver function, aiming to maximize tumor embolization while preserving healthy liver tissue. Microcatheters are strategically used for superselective catheterization into the tumor supply artery branch, enhancing the precision and efficacy of the embolization. (4) Post-embolization assessment: Following embolization, angiography is repeated to confirm the successful blockade of the tumor’s blood supply and assess the condition of the hepatic arteries’ remaining branches. Local ablation was a treatment that used physical or chemical methods to directly kill tumor tissue under image guidance, mainly including radiofrequency ablation (RFA), microwave ablation (MWA), and argon-helium cryoablation (AHC) in this study. Ultrasound, computed tomography (CT), and so on was used as image guidance for ablation treatment.

### Study endpoint and follow-up

The endpoint of this study was recurrence free survival (RFS), which referred to the time period from the combination treatment to the first recurrence or the last follow-up. Tumor recurrence was defined as the discovery of typical tumor manifestations through follow-up imaging examinations such as contrast-enhanced CT or magnetic resonance imaging (MRI) after treatment, or confirmed by pathological examination. Liver contrast-enhanced CT or MRI and laboratory examination were performed one month after treatment. Laboratory tests included blood routine, coagulation function, serum AFP levels, related enzymes and other indicators. Following that, regular follow-up appointments were conducted every 3 months for the first year, and starting from the second year, they were extended to every 6 months until recurrence or the last follow-up. The follow-up methods of this study included regular outpatient re-examination, inpatient re-examination, and telephone follow-up. The last follow-up date was January 1, 2023, with a median follow-up time of 29.05 months.

### Statistical analysis

All data analyses were conducted using R version 4.3.2 in this study, with P<0.05 considered statistically significant. Categorical variables were presented as number and proportion, and were compared using the χ^2^-test. Continuous variables were presented as mean ± standard deviation, and were compared using the Student’s t-test. Random survival forest (RSF), least absolute shrinkage and selection operator (LASSO) regression and multivariate Cox proportional hazards analysis were used to identify factors associated with RFS after HCC treatment. RSF was chosen for its ability to assess variable importance through survival trees, while LASSO regression facilitated the selection of relevant predictors by minimizing the prediction error and penalizing the absolute size of the regression coefficients. Hazard ratios (HR) and 95% confidence intervals (CI) were adopted to describe relevant risk factors in multivariate Cox analysis. Subsequently, a visual nomogram model was constructed based on the identified risk factors. We then evaluated the discrimination, calibration, and clinical application value of this model. Discrimination was assessed through the concordance index (C-index) and Area Under Curve (AUC) value. Calibration was evaluated using calibration curves, which compare the predicted probabilities of survival generated by the model with the actual observed survival rates. To determine the clinical application value of the nomogram, we conducted a decision curve analysis (DCA). DCA assesses the net benefit of using the model in clinical decision-making by considering the potential harms and benefits associated with different treatment thresholds. In addition, according to the scores generated by the nomogram, patients were categorized into three risk groups: low, intermediate, and high-risk groups. We employed the Kaplan-Meier method to plot the survival curves, and the Log-Rank test was used to assess whether there were statistically significant differences in recurrence rates among the different risk groups.

## Results

### Clinicopathological characteristics of the enrolled patients

This study ultimately included data from 255 patients and randomly assigned them to the training cohort (n=178) and validation cohort (n=77) in a 7:3 ratio. The training cohort was employed to identify risk factors and construct a nomogram prediction model, while the validation cohort was utilized to assess the model’s broad applicability. Through baseline characteristic comparison, it was confirmed that there was no statistically significant difference in the variables between the two cohorts (**Table 1**). The data presented included various parameters such as age, gender, tumor characteristics, and laboratory test results. The mean age was similar in both cohorts (58.02 ± 7.48 years old in the training cohort and 58.04 ± 7.03 years old in the validation cohort). And the gender distribution was also comparable: 132/178 (74.2%) male in the training cohort vs. 59/77 (76.6%) male in the validation cohort. In terms of tumor characteristics, the majority of patients in both cohorts had a single tumor: 116/178 (65.2%) in the training cohort and 54/77 (70.1%) in the validation cohort. The tumor size distribution was also similar, with the majority of tumors being ≤3cm in both cohorts. The laboratory test results such as Alb/Glb, GGT and uric acid also showed no significant differences between the two cohorts.

**Table 1:** Comparison of the clinicopathological characteristics between the training cohort and the validation cohort.

Characteristic	Training cohort(N=178)	Validation cohort(N=77)	P value
Age	58.02 ± 7.48	58.04 ± 7.03	0.982
Gender (male/female)	132(74.2%)/46(25.8%)	59(76.6%)/18(23.4%)	0.677
Hypertension (yes/no)	43(24.2%)/135(75.8%)	13(16.9%)/64(83.1%)	0.198
Diabetes (yes/no)	50(28.1%)/128(71.9%)	21(27.3%)/56(72.7%)	0.894
Smoking history (yes/no)	80(44.9%)/98(55.1%)	34(44.2%)/43(55.8%)	0.908
Drinking history (yes/no)	67(37.6%)/111(62.4%)	25(32.5%)/52(67.5%)	0.430
Tumor number (Single/multiple)	116(65.2%)/62(34.8%)	54(70.1%)/23(29.9%)	0.440
Tumor size (≤3cm/>3cm)	124(69.7%)/54(30.3%)	55(71.4%)/22(28.6%)	0.777
NLR	3.26 ± 2.68	3.37 ± 2.90	0.774
PLR	101.06 ± 48.78	111.28 ± 83.97	0.225
MLR	0.41 ± 0.21	0.42 ± 0.20	0.912
Hb	117.07 ± 18.43	118.47 ± 17.65	0.573
PLT	90.28 ± 44.66	91.30 ± 46.37	0.869
AST	35.71 ± 17.56	36.58 ± 15.35	0.708
ALT	30.23 ± 19.74	26.95 ± 15.20	0.195
AST/ALT	2.08 ± 9.44	1.53 ± 0.63	0.616
TP	62.47 ± 7.87	63.17 ± 7.36	0.509
Albumin	33.87 ± 5.24	33.56 ± 3.83	0.640
Globulin	29.30 ± 6.15	30.65 ± 6.41	0.113
Alb/Glb	1.21 ± 0.34	1.19 ± 0.28	0.650
GGT	74.81 ± 64.80	72.79 ± 78.07	0.83
GGT/ALT	2.82 ± 2.45	3.10 ± 3.14	0.448
ALP	101.52 ± 45.08	106.73 ± 41.87	0.387
Prealbumin	98.96 ± 41.23	92.93 ± 35.96	0.267
Uric acid	285.24 ± 107.61	259.62 ± 93.75	0.071
PT	13.61 ± 1.67	14.01 ± 1.80	0.088
INR	1.21 ± 0.15	1.25 ± 0.16	0.06
Fibrinogen	2.44 ± 0.85	2.43 ± 0.91	0.97
Fib/Alb	0.07 ± 0.03	0.07 ± 0.03	0.542
AFP	251.26 ± 848.10	138.49 ± 337.50	0.26

NLR, neutrophil to lymphocyte ratio; PLR, platelet to lymphocyte ratio; MLR, monocyte to lymphocyte ratio; Hb, hemoglobin; PLT, platelet; AST, aspartate aminotransferase; ALT, alanine aminotransferase; TP, total protein; Alb, albumin; Glb, globulin; GGT, gamma glutamyl transpeptidase; ALP, alkaline phosphatase; PT, prothrombin time; INR, international normalized ratio; Fib, fibrinogen; AFP, alpha-fetoprotein.

### Identifying the independent factors for RFS through RSF, LASSO and multivariate Cox regression

In this study, we initially employed an RSF model to screen for relevant variables that may impact RFS. Subsequently, we further refined the selection of variables through LASSO regression. Further screening was performed using multivariate Cox regression, identifying factors with a significance level (P < 0.05) as independent risk factors influencing RFS.

**Figure 2:**
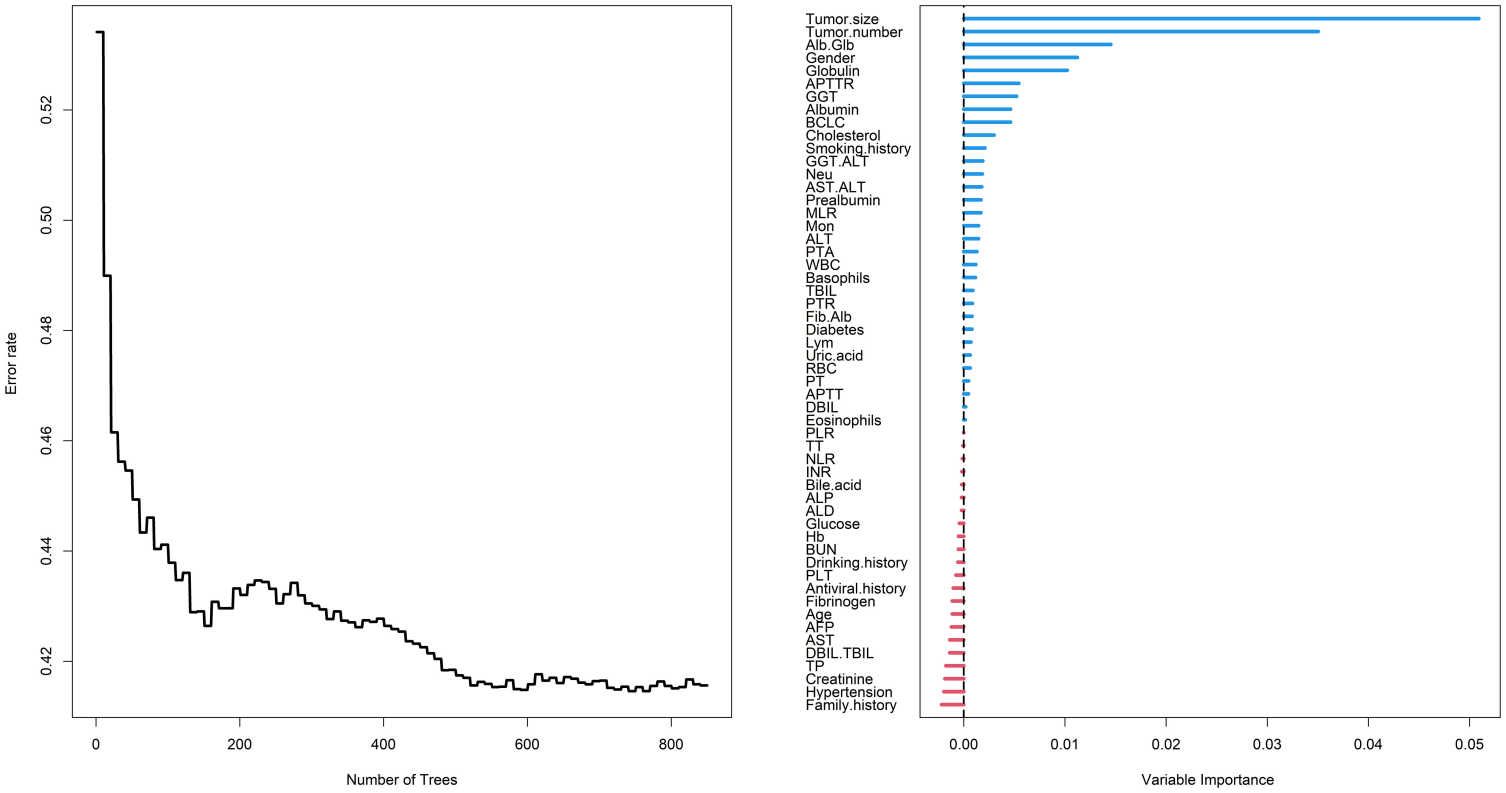
Random survival forest employed to screen risk factors for RFS. RFS, recurrence-free survival; Alb, albumin; Glb, globulin; APTTR, activated partial thromboplastin time ratio; GGT, gamma glutamyl transpeptidase; BCLC, Barcelona Clinic Liver Cancer; ALT, alanine aminotransferase; Neu, neutrophil; AST, aspartate aminotransferase; MLR, monocyte to lymphocyte ratio; Mon, monocyte; PTA, prothrombin time activity; WBC, white blood cell; TBIL, total bilirubin; PTR, prothrombin time ratio; Fib, fibrinogen; Lym, lymphocyte; RBC, red blood cell; PT, prothrombin time; APTT, activated partial thromboplastin time; DBIL, direct bilirubin; PLR, platelet to lymphocyte ratio; TT, thrombin time; NLR, neutrophil to lymphocyte ratio; INR, international normalized ratio; ALP, alkaline phosphatase; ALD, alcoholic liver disease; Hb, hemoglobin; PLT, platelet; AFP, alpha-fetoprotein; TBIL, total bilirubin; TP, total protein.

RSF is an improved algorithm of random forest (RF), primarily employed for analyzing survival data (12). Its mathematical principles are similar to RF, wherein a number of samples are randomly extracted from the original data using the bootstrap method. For each sample, a binary recursive survival tree is constructed, facilitating the selection of the most important variables associated with time and events. During the training process, the RSF algorithm inherently calculates the importance of each variable based on how much it contributes to the model’s predictive accuracy. This importance score is often referred to as VIMP (Variable Importance). If the Variable Importance (VIMP) computed by RSF is a positive value, it indicates that the variable can increase the accuracy of the predictive model; conversely, it will decrease the accuracy of the predictive model if the VIMP is negative (13). In the study, through parameter tuning, it was observed that the model’s error rate stabilized when constructing 850 trees. At the beginning, a total of 54 variables were included in the RSF, and ultimately 32 variables with positive VIMP were screened out as showing in **Figure 2**, including tumor size, tumor number, Alb/Glb, gender, globulin, activated partial thromboplastin time ratio (APTTR), GGT, albumin, BCLC, cholesterol, smoking history, GGT/ALT, Neu, AST/ALT, prealbumin, MLR, Mon, ALT, prothrombin time activity (PTA), WBC, basophils, TBIL, prothrombin time ratio (PTR), Fib/Alb, diabetes, Lym, uric acid, RBC, PT, activated partial thromboplastin time (APTT), DBIL and eosinophils.

**Figure 3:**
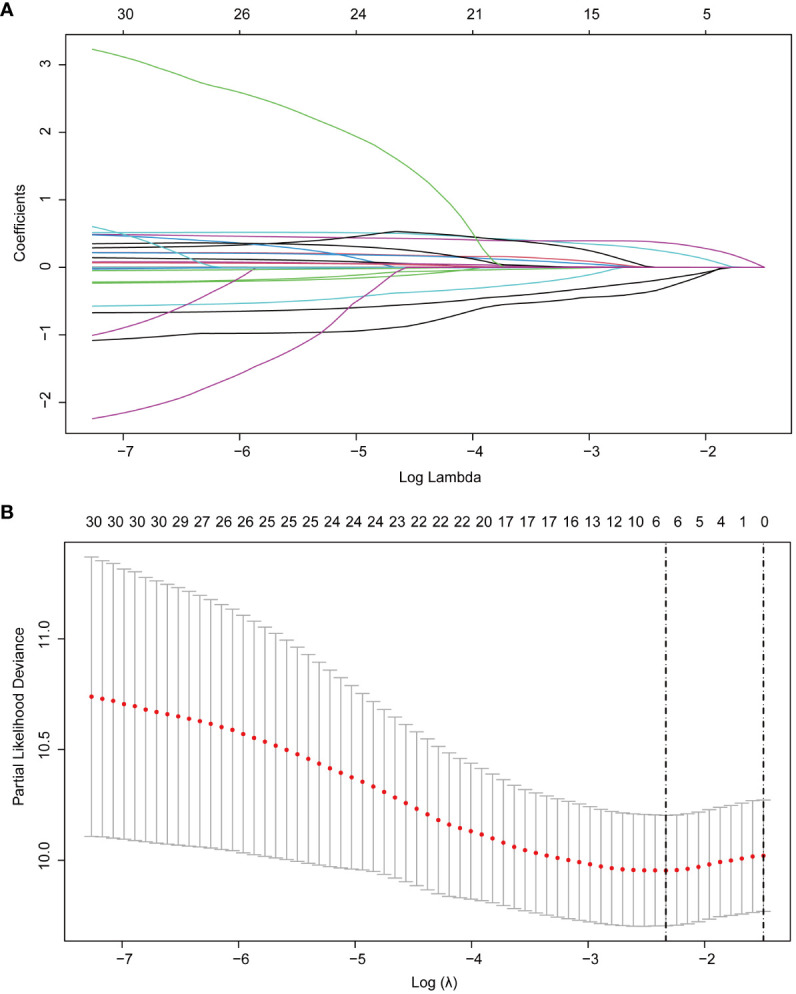
LASSO regression employed to further identified risk factors for RFS. **(A)** LASSO regression coefficients paths; **(B)** LASSO regression cross-validation curve. LASSO, least absolute shrinkage and selection operator; RFS, recurrence-free survival.

Then, based on the RSF results, in order to further improve the interpretability and generalization performance of the model, we introduced LASSO regression to select the most significant factors influencing RFS. LASSO regression is a regression method that streamlines the model by the L1 regularization term and continuously compressing the coefficients in multiple linear regression to avoid collinearity and overfitting (14, 15). In LASSO, the λ value is particularly important, related to the number of selected variables and the performance of the model. The optimal λ in LASSO regression reduces the number of variables by increasing the regularization strength until a balance between model fit and sparsity is achieved. This process automatically selects the most relevant variables for the model, improving both prediction accuracy and model simplicity, and it is calculated based on 10-fold cross-validation and minimization criteria. The optimal λ determined in this study was 0.0971096 (logλ= -2.3319), and 32 variables were reduced to 6 variables with non-zero coefficients as potential predictors in the training cohort, including gender, tumor number, tumor size, Alb/Glb, GGT and uric acid (**Figure 3**).

**Figure 4:**
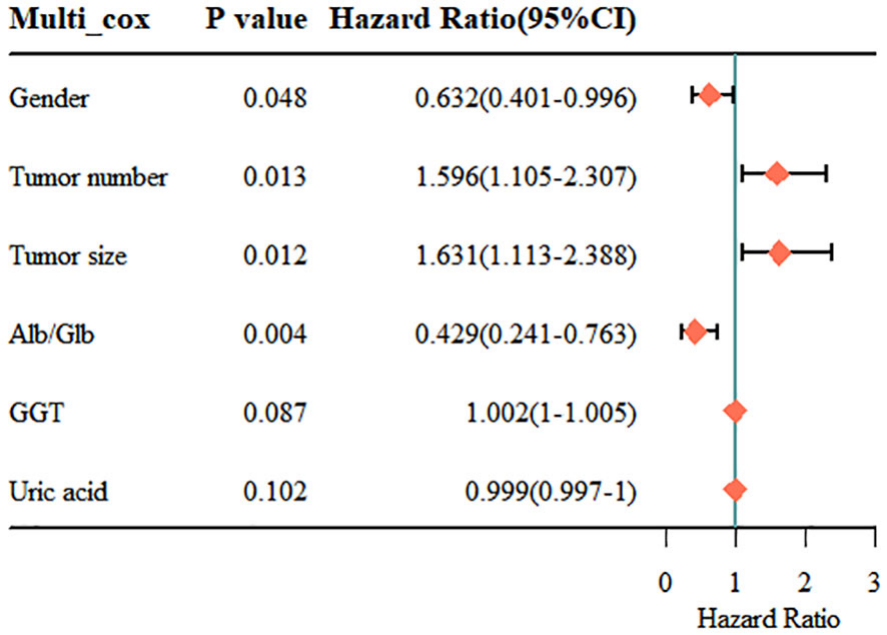
Results of the multivariate Cox regression analysis. Alb, albumin; Glb, globulin; GGT, gamma glutamyl transpeptidase.

Finally, we included the 6 non-zero coefficient variables screened by LASSO regression as potential risk factors into the multivariate Cox regression analysis. The results showed that four variables had P<0.05, so these 4 variables were all independent risk factors for RFS, including gender (HR: 0.632; 95% CI: 0.401 - 0.996; P=0.048), tumor number (HR: 1.596; 95% CI: 1.105 - 2.307; P=0.013), tumor size (HR: 1.631; 95% CI: 1.113 - 2.388; P=0.012) and Alb/Glb (HR: 0.429; 95% CI: 0.241 - 0.763; P=0.004). The results of multivariate Cox regression were displayed in the forest plot (**Figure 4**). Gender is an important risk factor as it may be indicative of hormonal or genetic differences that can affect cancer development and progression. Tumor number and size are key indicators of disease severity and aggressiveness; a higher tumor count or larger tumor size often correlates with a poorer prognosis. The Alb/Glb ratio provides insights into a patient’s nutritional status and liver function, which can significantly impact the body’s ability to fight disease and recover from treatment.

### Nomogram established for RFS based on the identified factors

**Figure 5:**
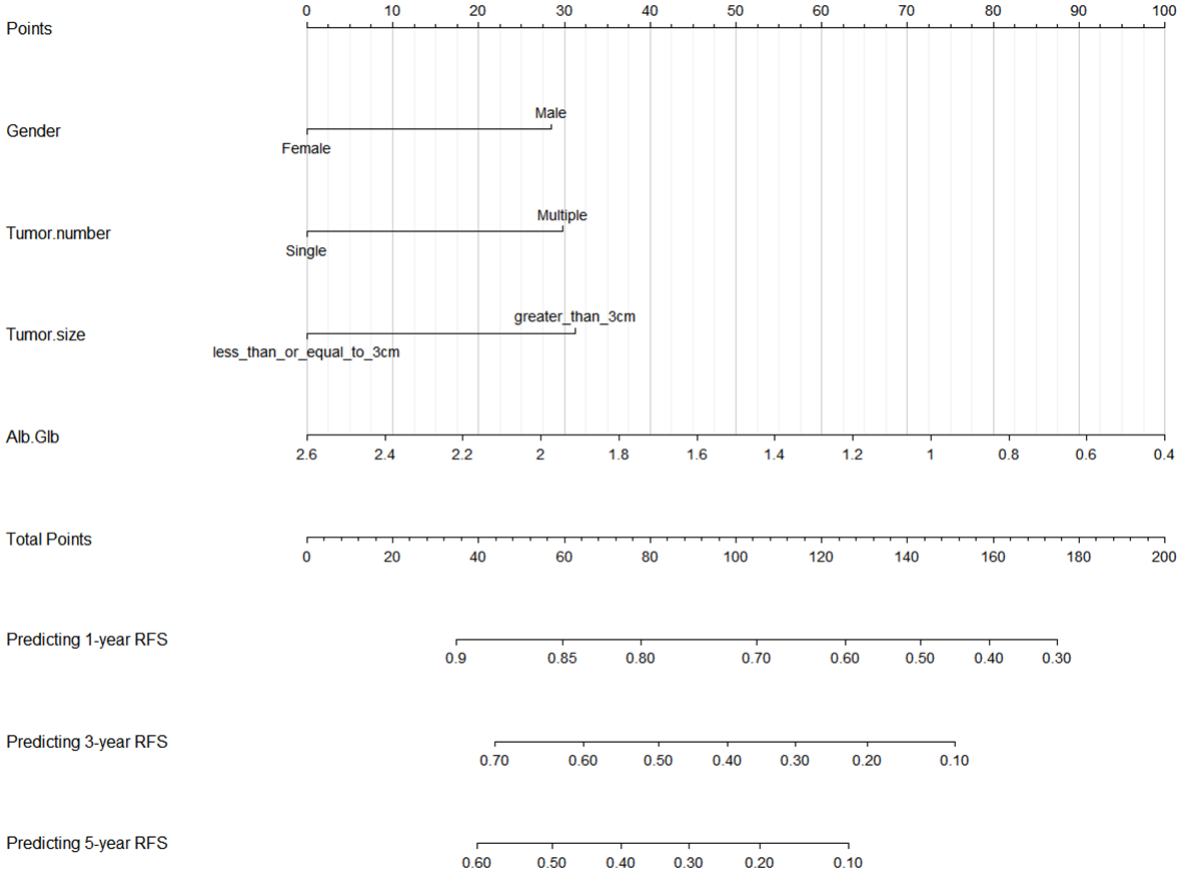
Nomogram for predicting 1-, 3-, and 5-year RFS in BCLC A/B HCC patients with Child-Pugh B cirrhosis after TACE combined with ablation therapy. RFS, recurrence-free survival; BCLC, Barcelona Clinic Liver Cancer; HCC, hepatocellular carcinoma; TACE, transcatheter arterial chemoembolization; Alb, albumin; Glb, globulin.

We established a nomogram using the four independent risk factors (gender, tumor number, tumor size and Alb/Glb) screened out by RSF, LASSO and multivariate Cox regression analysis for predicting recurrence after TACE combined with ablation therapy in BCLC A/B HCC patients with Child-Pugh B cirrhosis (**Figure 5**). The nomogram consisted of four parts: points, predictive factors (four independent risk factors), total points and predicted probability. The points corresponding to each predictive factor of the patient were added together to obtain the total points. The values on the 1-, 3-, and 5-year RFS coordinate axes corresponding to the total points were the predicted probabilities of 1-, 3-, and 5-year RFS for patients. The performance of the model would be evaluated in the following section, including C-index, AUC, calibration curves and DCA curves.

### Predictive performance of the nomogram in training cohort

In order to ensure the scientific credibility of the research results, we must conduct a detailed evaluation of the performance of the nomogram, generally including discrimination, calibration, and clinical utility. Discrimination was evaluated by C-index and AUC, calibration curves and DCA curves were employed to measure calibration and clinical utility of the nomogram, respectively.

**Figure 6:**
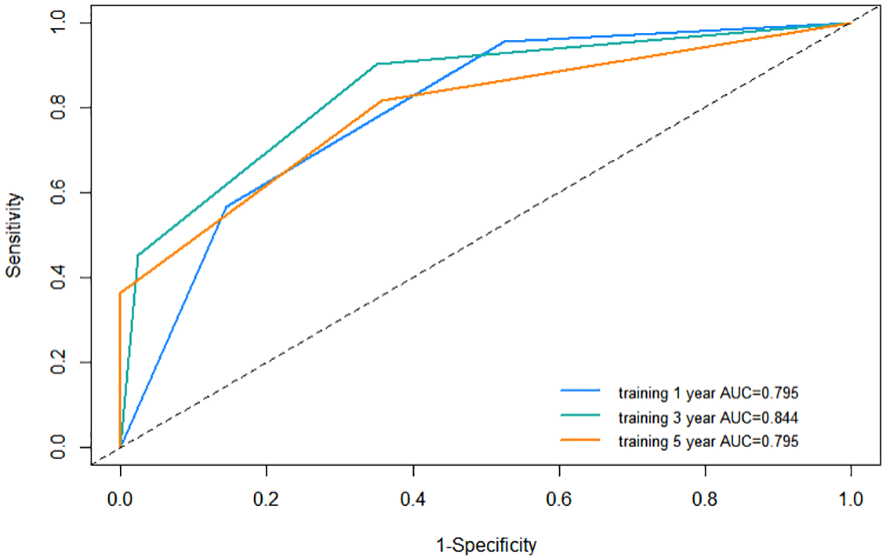
Receiver operating characteristic (ROC) curve of the nomogram in the training cohort. AUC, area under the curve.

Firstly, we assessed the discrimination ability of nomogram using the C-index and AUC value. The value range of C-index is 0.5~1. The larger the value of C-index, the higher the accuracy of the nomogram. The C-index for the nomogram within the training cohort exhibited a commendable value of 0.744 (95% CI: 0.703–0.785), underscoring its adeptness in discriminating between patients who would experience recurrence and those who would not. The receiver operating characteristic curve (ROC) is another commonly used tool to evaluate the discrimination of nomogram. The area enclosed by the ROC curve and the horizontal and vertical coordinates is called AUC, and its value range is also 0.5~1. In the training cohort, the AUC values for 1-year, 3-year, and 5-year RFS were 0.795, 0.844, and 0.795, respectively, providing additional support for its outstanding discrimination ability (**Figure 6**).

**Figure 7:**
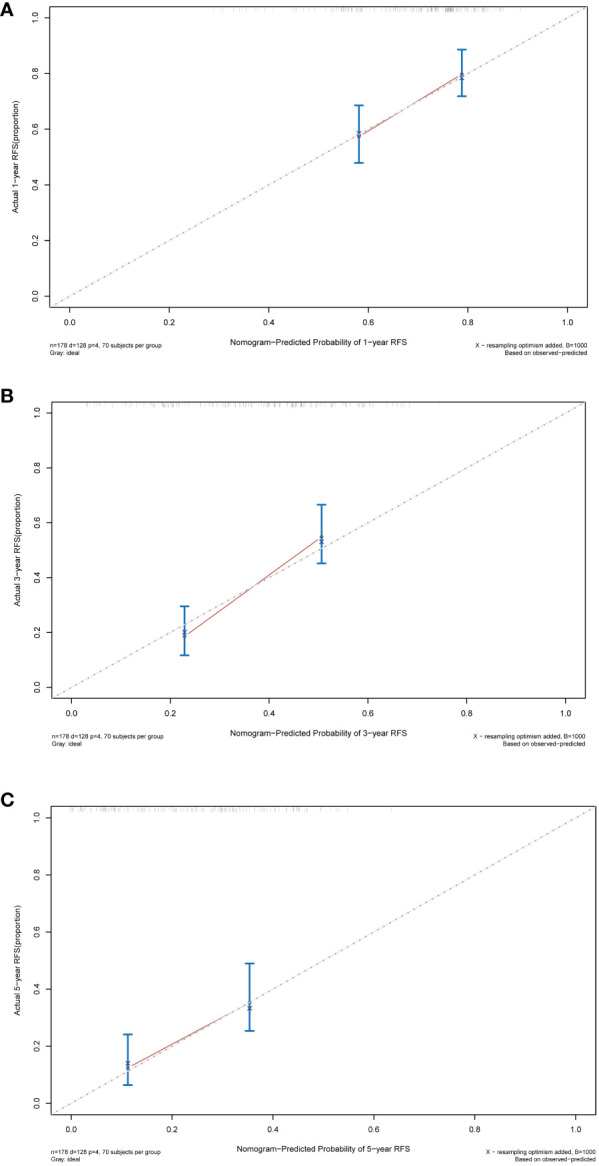
Calibration curves of the nomogram in the training cohort. **(A)** Calibration curve for predicting 1-year RFS. **(B)** Calibration curve for predicting 3-year RFS. **(C)** Calibration curve for predicting 5-year RFS. RFS, recurrence-free survival.

Following this, we plotted calibration curves to evaluate the nomogram’s accuracy by comparing the predicted probabilities against the actual observed frequencies. The dashed line represents an ideal curve, while the solid line represents the predictive ability of the nomogram in this study. The closer the distance between the two is, the more accurate the actual prediction ability will be. By observing the 1-year, 3-year, and 5-year calibration curves in the training cohort, it can be found that the calibration curves of this study was relatively close to the dashed line, suggesting that the nomogram had a good predictive accuracy between the actual probability and predicted probability (**Figure 7**).

**Figure 8:**
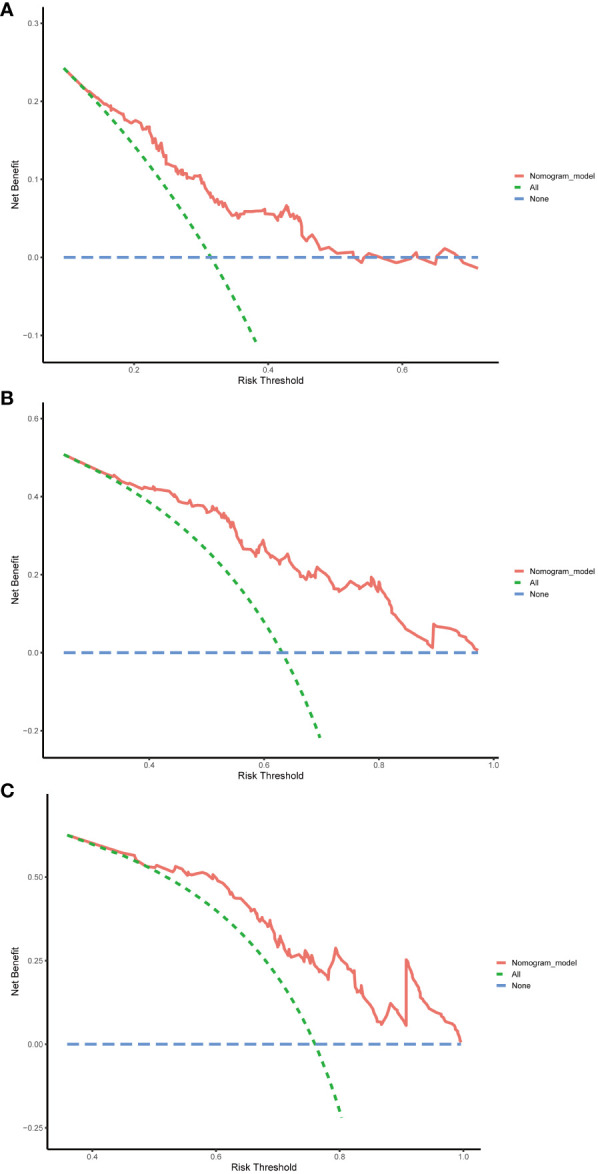
Decision curve analysis (DCA) of the nomogram in the training cohort. **(A)** DCA curve for predicting 1-year RFS. **(B)** DCA curve for predicting 3-year RFS. **(C)** DCA curve for predicting 5-year RFS. RFS, recurrence-free survival.

Finally, DCA was employed to assess the clinical utility of the nomogram. The horizontal axis of the curve represents threshold probabilities, while the vertical axis represents the net benefit. The net benefit when all patients do not relapse and no clinical intervention is performed is represented by the None line. Conversely, the net benefit when all patients relapse and clinical intervention is performed is represented by the All line. When the net benefit rate corresponding to a probability threshold is located on the upper right side of the All line and None line (the net benefit rates corresponding to each threshold probability are concatenated into a red line), it indicates that the predictive model has good clinical utility. In our study, the 1-year, 3-year, and 5-year DCA curves of the training cohort showed that within a certain threshold probability range, the probability of patient benefiting from this nomogram was greater than the two extreme cases, that is, the net benefit was positive, indicating that the model has high clinical utility (**Figure 8**).

### Predictive performance of the nomogram in validation cohort

The nomogram’s performance was further evaluated in the validation cohort, aiming at assessing the universal applicability of the nomogram. In the validation cohort, the nomogram exhibited a robust discrimination ability with a C-index of 0.724 (95% CI: 0.644–0.804), and corresponding AUC values of 0.758, 0.695, and 0.638 for predicting 1-year, 5-year, and 10-year RFS, respectively (**Supplementary Figure S1**). The calibration curves also demonstrated good consistency between the probabilities generated by the nomogram and the corresponding actual probabilities for the 1-year, 3-year, and 5-year RFS (**Supplementary Figure S2**). The DCA curve in the validation cohort also showed that the nomogram can bring good net benefits for predicting the 1-year, 3-year, and 5-year RFS after TACE combined with ablation in BCLC A/B HCC patients with Child-Pugh B cirrhosis and has high clinical effectiveness (**Supplementary Figure S3**).

### Kaplan-Meier curves of RFS for patients in low, intermediate, and high-risk groups categorized by the nomogram scores

**Figure 9:**
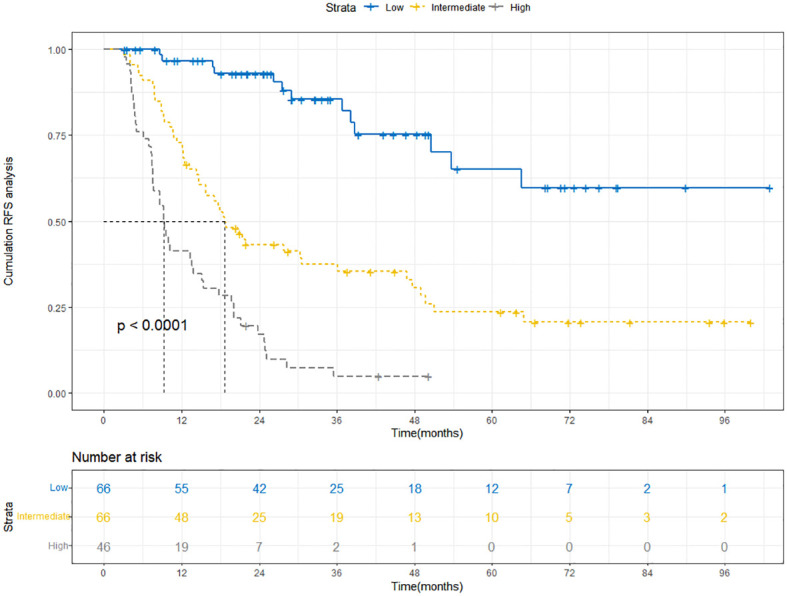
Kaplan-Meier curves depict RFS in the training cohort based on nomogram-derived risk groups. RFS, recurrence-free survival.

The Kaplan-Meier curve is a commonly used survival analysis method that is often used to evaluate the survival status of patients. This section focused on classifying patients into low, intermediate, and high-risk groups based on nomogram scores, and explored the RFS of these groups through Kaplan-Meier curves. According to the Kaplan-Meier curve from the training cohort (**Figure 9**), we observed that the curve for the low-risk group remained relatively stable, indicating a favorable survival rate among patients. In contrast, the curve for the high-risk group exhibited a significant downward trend, suggesting a lower survival rate. This implied that the nomogram was, to some extent, effective in predicting patients’ RFS and facilitating risk stratification to identify high-risk patients. The Kaplan-Meier curve of the validation cohort further strengthened our observation of the survival differences between the high-risk and low-risk groups (**Supplementary Figure S4**).

## Discussion

In recent years, TACE combined with local ablation therapy has gradually attracted attention as an emerging treatment method. ESMO Clinical Practice Guidelines (2018) recommend local ablation for stage A and TACE for stage B patients (16). TACE slows tumor growth by cutting off blood supply and delivering chemo locally, allowing repeated treatments (17), albeit with challenges to complete cure (18). Local ablation is minimally invasive, offering survival rates akin to surgery, but its post-treatment recurrence remains an issue (19–21). Reports suggest the combined therapy surpasses either method alone for HCC patients (11). In HCC patients with BCLC stage A/B, combination therapy is now considered a common treatment method. Patients of this kind often present with concomitant cirrhosis (22). However, it must be acknowledged that there is a notable lack of research on the prognosis associated with the combination therapy in patients with BCLC stage A/B HCC accompanied by Child-Pugh B cirrhosis. Our study has, for the first time, bridged the gap in this particular field.

Our study employed a multi-level variable-screening method, including RSF, LASSO regression, and multivariate Cox regression, to gain a deeper understanding of the key factors affecting RFS in BCLC A/B HCC patients with Child-Pugh B cirrhosis after treatment. Through these analyses, we successfully screened out four key parameters with significant influence, namely gender, tumor number, tumor size, and Alb/Glb ratio. Gender plays a certain role in the prognosis of HCC patients. Previous studies have shown that female patients may have better prognosis in HCC compared to male patients (23, 24). Hormones, specifically androgens and estrogens, may have different effects on HCC. Androgen could potentially stimulate the development of HCC, whereas estrogen may exert a protective effect against HCC (25). Estrogen can inhibit the proliferation of liver cells and promote apoptosis, thereby suppressing the development of HCC (26). It can also restrict liver inflammation and fibrosis, inhibiting the progression of HCC. However, androgen may directly or indirectly regulate virus gene expression and host immune responses to elevate viral replication levels, thereby promoting the HCC development (27). In addition, some genes on the Y chromosome may play a role in the occurrence and aggressiveness of HCC in males (28). The number and size of tumors are also critical indicators for assessing the prognosis of HCC patients. In clinical practice, a single smaller tumor is typically more amenable to treatment, and the prognosis is relatively favorable. Conversely, multiple or larger tumors may pose more complex treatment challenges, leading to a relatively poorer prognosis. Tumor size is intricately linked to genetic alterations and pathological characteristics. Larger HCC tumor sizes, by their very nature, serve as potent incentives of invasive behavior (29). This heightened aggressiveness, in turn, contributes to an increased likelihood of recurrence and metastasis.

Contrary to tumor number and tumor size, the decrease in Alb/Glb ratio is often closely related to poor prognosis in HCC patients (30, 31). It serves as a measure of the proportional levels of albumin and globulin, providing insights into liver function, nutritional status and inflammatory conditions. Albumin, a crucial protein synthesized by the liver, serves as an indicator of liver function. Patients with HCC often experience impaired liver function, leading to a decrease in albumin levels (32). On the other hand, globulin is a vital component of the immune system, and its levels may increase in response to factors such as inflammation, which is a risk factor for inducing HCC (33). Albumin can also reflect the nutritional status of the patient, and a decrease in albumin levels indicates systemic malnutrition, which can lead to poor prognosis in HCC patients (34).

Despite the encouraging predictive performance demonstrated by the established nomogram in our study, it is crucial to acknowledge and address several limitations inherent in our study. Firstly, due to the specific focus of our study on a distinct population with unique characteristics, only 255 patients were finally included in the analysis, despite the full efforts of our group members in data collection. It is essential to recognize that the relatively modest sample size might impact the generalizability and robustness of our findings. We anticipate the inclusion of more suitable patients in future studies. Secondly, the lack of external validation is another limitation of our study. By testing our findings in different populations and settings in the future, we can better understand how they apply to real-world scenarios and inform practical decision-making. Thirdly, our study predominantly focused on clinical and pathological variables. The exclusion of comprehensive exploration of underlying biological factors, such as specific genetic markers or molecular signatures, may limit the depth of our predictive model. Moreover, our study did not extensively consider external environmental factors that might contribute to HCC risk. Factors such as pollution, occupational exposures, and socio-economic conditions could play a role in HCC development and should be addressed in future studies to provide a more comprehensive risk assessment.

## Conclusion

Our study has successfully developed and rigorously validated a nomogram incorporating four pivotal clinical variables, which were meticulously screened through RSF, LASSO, and multivariate Cox regression analyses. This nomogram exhibits remarkable predictive accuracy, as evidenced by the C-index values (0.744 in the training cohort and 0.724 in the validation cohort), promising AUC values, calibration curves, and DCA curves in both cohorts. Its potential clinical impact is profound, as it offers personalized predictions of 1-year, 3-year, and 5-year RFS, and it can promptly identify high-risk populations. This is crucial for planning long-term treatment and follow-up strategies.

## Data availability statement

The original contributions presented in the study are included in the article/**Supplementary Material**. Further inquiries can be directed to the corresponding authors.

## Ethics statement

The studies involving humans were approved by Ethics Committee of Beijing You’an Hospital, affiliated with Capital Medical University. The studies were conducted in accordance with the local legislation and institutional requirements. The ethics committee/institutional review board waived the requirement of written informed consent for participation from the participants or the participants’ legal guardians/next of kin because The necessity for securing written informed consent for data publication was waived, as the identities of the patients involved were maintained in strict anonymity.

## Author contributions

WQ: Conceptualization, Data curation, Formal Analysis, Methodology, Writing – original draft, Writing – review & editing. SS: Conceptualization, Formal Analysis, Methodology, Writing – original draft, Writing – review & editing. YX: Conceptualization, Writing – original draft, Writing – review & editing, Data curation. MH: Conceptualization, Writing – review & editing, Methodology, Project administration, Supervision. RJ: Conceptualization, Project administration, Supervision, Resources, Writing – review & editing. CH: Conceptualization, Funding acquisition, Methodology, Project administration, Supervision, Writing – review & editing.

## Supplementary material

Supplementary Figure 1 Receiver operating characteristic curve (ROC) of the nomogram in the validation cohort. AUC, area under the curve.

Supplementary Figure 2 Calibration curves of the nomogram in the validation cohort. **(A)** Calibration curve for predicting 1-year RFS. **(B)** Calibration curve for predicting 3-year RFS. **(C)** Calibration curve for predicting 5-year RFS. RFS, recurrence-free survival.

Supplementary Figure 3 Decision curve analysis (DCA) of the nomogram in the validation cohort. **(A)** DCA curve for predicting 1-year RFS. **(B)** DCA curve for predicting 3-year RFS. **(C)** DCA curve for predicting 5-year RFS. RFS, recurrence-free survival.

Supplementary Figure 4 Kaplan-Meier curves depict RFS in the validation cohort based on nomogram-derived risk groups. RFS, recurrence-free survival.

## References

[1] Rumgay H Arnold M Ferlay J Lesi O Cabasag CJ Vignat J. Global burden of primary liver cancer in 2020 and predictions to 2040. J Hepatol. (2022) 77:1598–606. doi: 10.1016/j.jhep.2022.08.021 PMC9670241 36208844

[2] Ducreux M Abou-Alfa GK Bekaii-Saab T Berlin J Cervantes A de Baere T. Current expert opinion and recommendations derived from the 24th ESMO/World Congress on Gastrointestinal Cancer, Barcelona, 2022. ESMO Open. (2023) 8:101567. doi: 10.1016/j.esmoop.2023.101567 37263081 PMC10245111

[3] Brown ZJ Tsilimigras DI Ruff SM Mohseni A Kamel IR Cloyd JM. Management of hepatocellular carcinoma: A review. JAMA Surg. (2023) 158:410–20. doi: 10.1001/jamasurg.2022.7989 36790767

[4] Lin J Zhang H Yu H Bi X Zhang W Yin J. Epidemiological characteristics of primary liver cancer in mainland China from 2003 to 2020: A representative multicenter study. Front Oncol. (2022) 12:906778. doi: 10.3389/fonc.2022.906778 35800051 PMC9253580

[5] Zheng R Qu C Zhang S Zeng H Sun K Gu X. Liver cancer incidence and mortality in China: Temporal trends and projections to 2030. Chin J Cancer Res. (2018) 30:571–9. doi: 10.21147/j.issn.1000-9604.2018.06.01 PMC6328503 30700925

[6] Tsoris A Marlar CA. Use of the child pugh score in liver disease. In: Treasure Island (FL). StatPearls Publishing Copyright © 2023, StatPearls Publishing LLC (2023). 31194448

[7] Watanabe Y Aikawa M Kato T Takase K Watanabe Y Okada K. Influence of Child-Pugh B7 and B8/9 cirrhosis on laparoscopic liver resection for hepatocellular carcinoma: a retrospective cohort study. Surg Endosc. (2023) 37:1316–33. doi: 10.1007/s00464-022-09677-x PMC9540096 36203111

[8] Allaire M Goumard C Lim C Le Cleach A Wagner M Scatton O. New frontiers in liver resection for hepatocellular carcinoma. JHEP Rep. (2020) 2:100134. doi: 10.1016/j.jhepr.2020.100134 32695968 PMC7360891

[9] Chen W Zheng R Baade PD Zhang S Zeng H Bray F. Cancer statistics in China, 2015. CA Cancer J Clin. (2016) 66:115–32. doi: 10.3322/caac.21338 26808342

[10] Jiang FQ Lu W Yang C Du P Ma JP Yang J. Curative effect of transcatheter arterial chemoembolization combined with radiofrequency ablation in treating hepatic cell carcinoma and its effect on serum markers. Cancer biomark. (2017) 20:17–22. doi: 10.3233/CBM-160508 28582848

[11] Jiang C Cheng G Liao M Huang J. Individual or combined transcatheter arterial chemoembolization and radiofrequency ablation for hepatocellular carcinoma: a time-to-event meta-analysis. World J Surg Oncol. (2021) 19:81. doi: 10.1186/s12957-021-02188-4 33741001 PMC7980330

[12] Pickett KL Suresh K Campbell KR Davis S Juarez-Colunga E. Random survival forests for dynamic predictions of a time-to-event outcome using a longitudinal biomarker. BMC Med Res Methodol. (2021) 21:216. doi: 10.1186/s12874-021-01375-x 34657597 PMC8520610

[13] Lezcano-Valverde JM Salazar F León L Toledano E Jover JA Fernandez-Gutierrez B. Development and validation of a multivariate predictive model for rheumatoid arthritis mortality using a machine learning approach. Sci Rep. (2017) 7:10189. doi: 10.1038/s41598-017-10558-w 28860558 PMC5579234

[14] Yang C Delcher C Shenkman E Ranka S. Machine learning approaches for predicting high cost high need patient expenditures in health care. BioMed Eng Online. (2018) 17:131. doi: 10.1186/s12938-018-0568-3 30458798 PMC6245495

[15] Dai P Chang W Xin Z Cheng H Ouyang W Luo A. Retrospective study on the influencing factors and prediction of hospitalization expenses for chronic renal failure in China based on random forest and LASSO regression. Front Public Health. (2021) 9:678276. doi: 10.3389/fpubh.2021.678276 34211956 PMC8239170

[16] Vogel A Cervantes A Chau I Daniele B Llovet JM Meyer T. Hepatocellular carcinoma: ESMO Clinical Practice Guidelines for diagnosis, treatment and follow-up. Ann Oncol. (2018) 29:iv238–iv55. doi: 10.1093/annonc/mdy308 30285213

[17] Zhou J Sun H Wang Z Cong W Zeng M Zhou W. Guidelines for the diagnosis and treatment of primary liver cancer (2022 edition). Liver Cancer. (2023) 12:405–44. doi: 10.1159/000530495 PMC10601883 37901768

[18] Li J Kong M Yu G Wang S Shi Z Han H. Safety and efficacy of transarterial chemoembolization combined with tyrosine kinase inhibitors and camrelizumab in the treatment of patients with advanced unresectable hepatocellular carcinoma. Front Immunol. (2023) 14:1188308. doi: 10.3389/fimmu.2023.1188308 37545497 PMC10401037

[19] Lee J Jin YJ Shin SK Kwon JH Kim SG Suh YJ. Surgery versus radiofrequency ablation in patients with Child- Pugh class-A/single small (≤3 cm) hepatocellular carcinoma. Clin Mol Hepatol. (2022) 28:207–18. doi: 10.3350/cmh.2021.0294 PMC9013608 34814239

[20] Takigawa A Sakamori R Tahata Y Yoshioka T Yamada R Kodama T. Prediction model for intrahepatic distant recurrence after radiofrequency ablation for primary hepatocellular carcinoma 2 cm or smaller. Dig Dis Sci. (2022) 67:5704–11. doi: 10.1007/s10620-022-07455-2 35353331

[21] Facciorusso A Del Prete V Antonino M Crucinio N Neve V Di Leo A. Post-recurrence survival in hepatocellular carcinoma after percutaneous radiofrequency ablation. Dig Liver Dis. (2014) 46:1014–9. doi: 10.1016/j.dld.2014.07.012 25085684

[22] Inchingolo R Posa A Mariappan M Spiliopoulos S. Locoregional treatments for hepatocellular carcinoma: Current evidence and future directions. World J Gastroenterol. (2019) 25:4614–28. doi: 10.3748/wjg.v25.i32.4614 PMC6718039 31528090

[23] Lai MW Chu YD Lin CL Chien RN Yeh TS Pan TL. Is there a sex difference in postoperative prognosis of hepatocellular carcinoma? BMC Cancer. (2019) 19:250. doi: 10.1186/s12885-019-5453-3 30894157 PMC6425676

[24] Rich NE Murphy CC Yopp AC Tiro J Marrero JA Singal AG. Sex disparities in presentation and prognosis of 1110 patients with hepatocellular carcinoma. Aliment Pharmacol Ther. (2020) 52:701–9. doi: 10.1111/apt.15917 PMC7655123 32598091

[25] Montella M D'Arena G Crispo A Capunzo M Nocerino F Grimaldi M. Role of sex hormones in the development and progression of hepatitis B virus-associated hepatocellular carcinoma. Int J Endocrinol. (2015) 2015:854530. doi: 10.1155/2015/854530 26491442 PMC4600563

[26] Guo Y Wu G Yi J Yang Q Jiang W Lin S. Anti-hepatocellular carcinoma effect and molecular mechanism of the estrogen signaling pathway. Front Oncol. (2021) 11:763539. doi: 10.3389/fonc.2021.763539 35096574 PMC8789654

[27] Ma WL Lai HC Yeh S Cai X Chang C. Androgen receptor roles in hepatocellular carcinoma, fatty liver, cirrhosis and hepatitis. Endocr Relat Cancer. (2014) 21:R165–82. doi: 10.1530/ERC-13-0283 PMC4165608 24424503

[28] Kido T Lau YC. The Y-linked proto-oncogene TSPY contributes to poor prognosis of the male hepatocellular carcinoma patients by promoting the pro-oncogenic and suppressing the anti-oncogenic gene expression. Cell Biosci. (2019) 9:22. doi: 10.1186/s13578-019-0287-x 30867900 PMC6399826

[29] Pawlik TM Delman KA Vauthey JN Nagorney DM Ng IO Ikai I. Tumor size predicts vascular invasion and histologic grade: Implications for selection of surgical treatment for hepatocellular carcinoma. Liver Transpl. (2005) 11:1086–92. doi: 10.1002/(ISSN)1527-6473 16123959

[30] Deng Y Pang Q Miao RC Chen W Zhou YY Bi JB. Prognostic significance of pretreatment albumin/globulin ratio in patients with hepatocellular carcinoma. Onco Targets Ther. (2016) 9:5317–28. doi: 10.2147/OTT PMC5005008 27601923

[31] Utsumi M Kitada K Tokunaga N Narusaka T Hamano R Miyasou H. Preoperative albumin-to-globulin ratio predicts prognosis in hepatocellular carcinoma: A cohort study including non-hepatitis virus-infected patients. Dig Surg. (2021) 38:307–15. doi: 10.1159/000518307 34515102

[32] Paar M Fengler VH Rosenberg DJ Krebs A Stauber RE Oettl K. Albumin in patients with liver disease shows an altered conformation. Commun Biol. (2021) 4:731. doi: 10.1038/s42003-021-02269-w 34127764 PMC8203801

[33] Zhang W Zhangyuan G Wang F Zhang H Yu D Wang J. High preoperative serum globulin in hepatocellular carcinoma is a risk factor for poor survival. J Cancer. (2019) 10:3494–500. doi: 10.7150/jca.29499 PMC6603401 31293654

[34] Gupta D Lis CG. Pretreatment serum albumin as a predictor of cancer survival: a systematic review of the epidemiological literature. Nutr J. (2010) 9:69. doi: 10.1186/1475-2891-9-69 21176210 PMC3019132

