# Supplementary figures and images for: Nomogram for predicting post-therapy recurrence in BCLC A/B hepatocellular carcinoma with Child-Pugh B cirrhosis
Source: Front Immunol. 2024 May 10;15:1369988. doi: 10.3389/fimmu.2024.1369988 (PMC11116566; doi:10.3389/fimmu.2024.1369988)

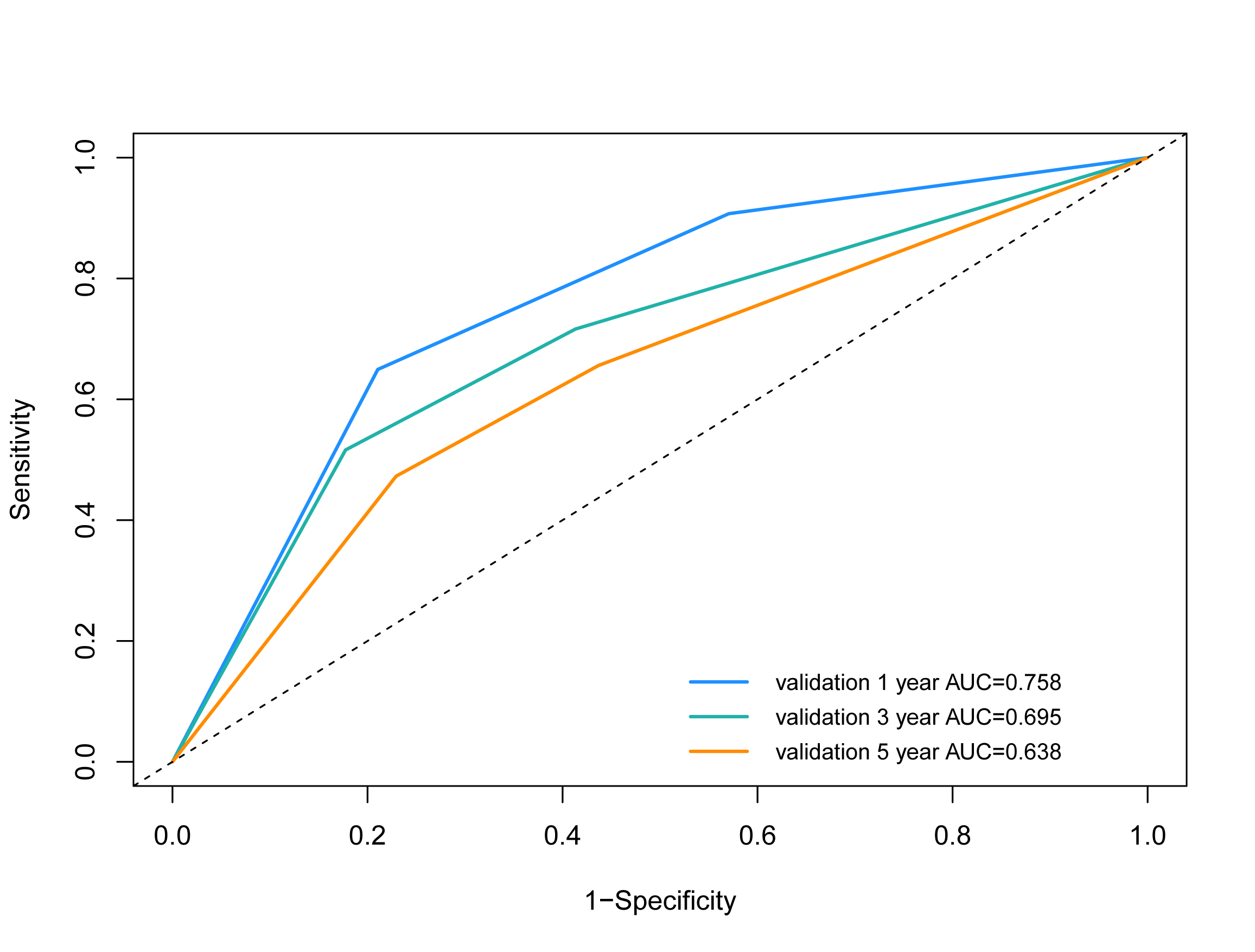

Supplement: Supplementary Figure 1 — Receiver operating characteristic curve (ROC) of the nomogram in the validation cohort. AUC, area under the curve. [file Image_1.tif]

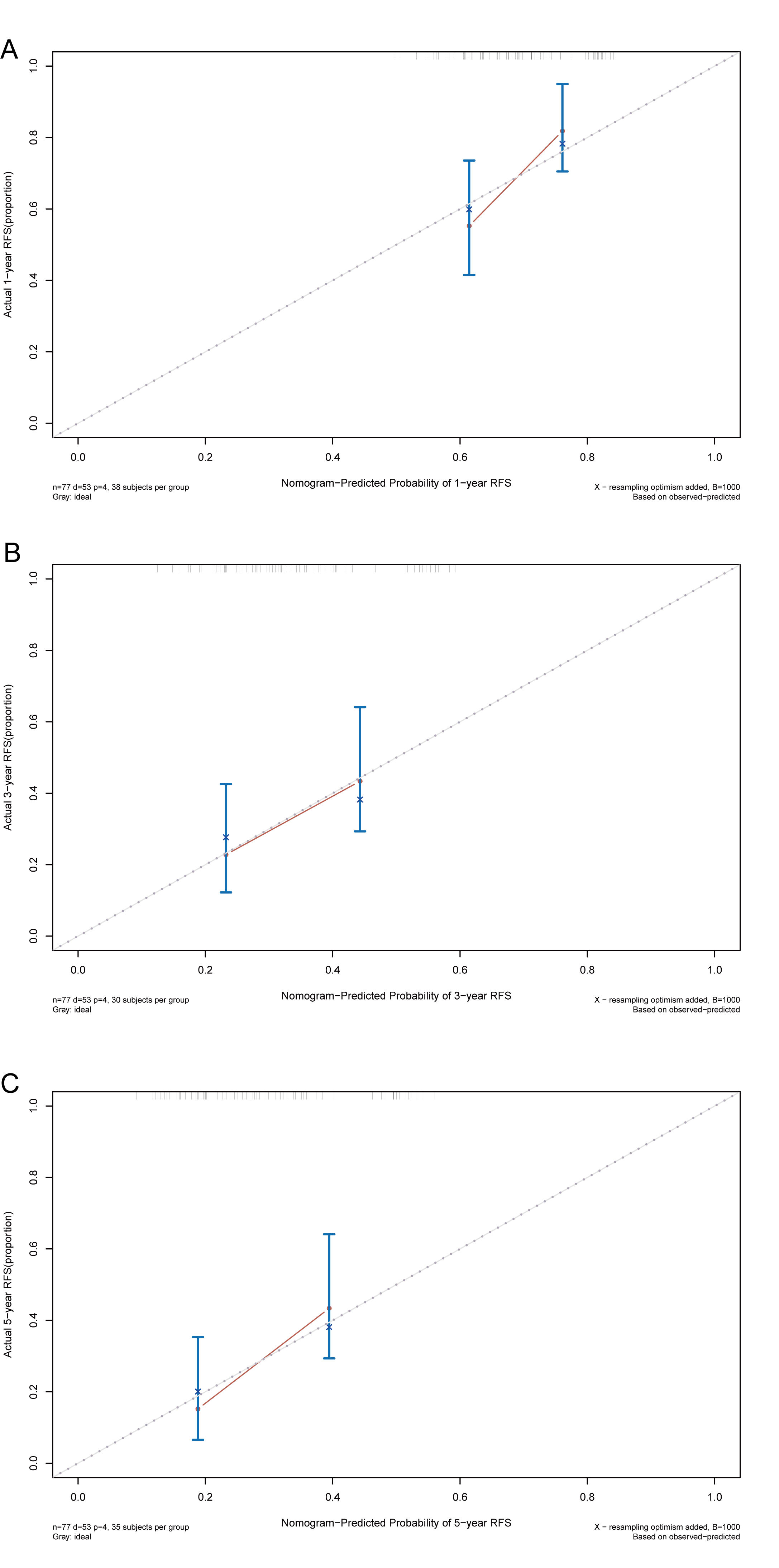

Supplement: Supplementary Figure 2 — Calibration curves of the nomogram in the validation cohort. (A) Calibration curve for predicting 1-year RFS. (B) Calibration curve for predicting 3-year RFS. (C) Calibration curve for predicting 5-year RFS. RFS, recurrence-free survival. [file Image_2.tif]

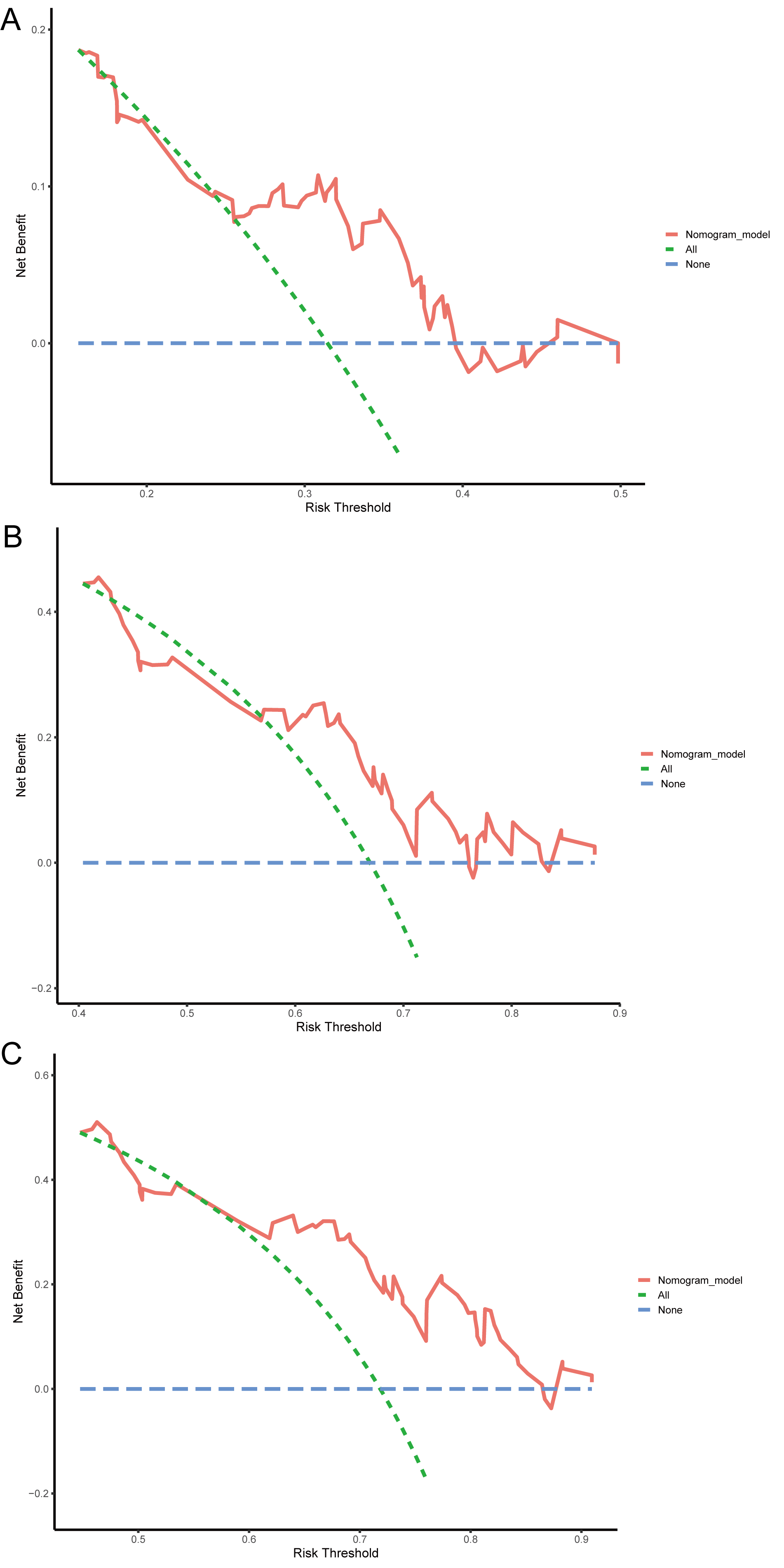

Supplement: Supplementary Figure 3 — Decision curve analysis (DCA) of the nomogram in the validation cohort. (A) DCA curve for predicting 1-year RFS. (B) DCA curve for predicting 3-year RFS. (C) DCA curve for predicting 5-year RFS. RFS, recurrence-free survival. [file Image_3.tif]

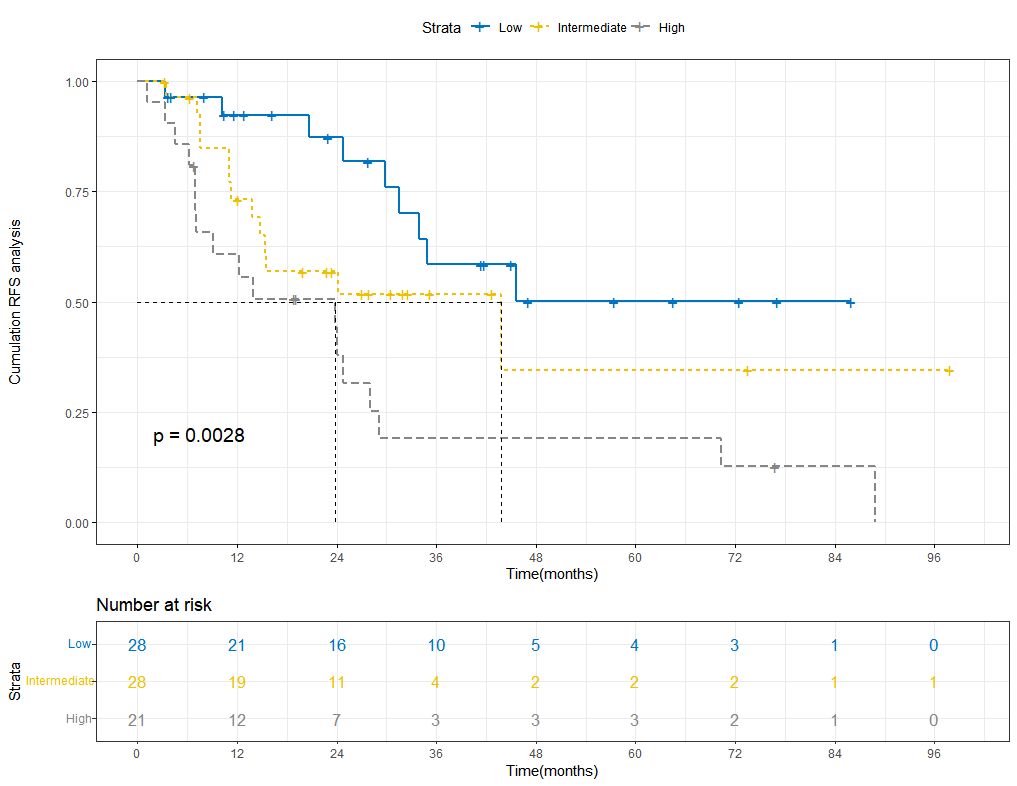

Supplement: Supplementary Figure 4 — Kaplan-Meier curves depict RFS in the validation cohort based on nomogram-derived risk groups. RFS, recurrence-free survival. [file Image_4.tiff]
